# Can asymmetric post‐translational modifications regulate the behavior of STAT3 homodimers?

**DOI:** 10.1096/fba.2019-00049

**Published:** 2020-01-27

**Authors:** Ricardo Letra‐Vilela, Beatriz Cardoso, Catarina Silva‐Almeida, Ana Maia Rocha, Fernanda Murtinheira, Joana Branco‐Santos, Carmen Rodriguez, Vanesa Martin, Mariana Santa‐Marta, Federico Herrera

**Affiliations:** ^1^ Cell Structure and Dynamics Laboratory Instituto de Tecnologia Quimica e Biologica (ITQB‐NOVA) Universidade Nova de Lisboa Oeiras Portugal; ^2^ Cell Structure and Dynamics Laboratory Faculdade de Ciências Universidade de Lisboa Lisbon Portugal; ^3^ Instituto Universitario de Oncología del Principado de Asturias (IUOPA) and Departamento de Morfología y Biología Celular Facultad de Medicina University of Oviedo Oviedo Spain

**Keywords:** acetylation, bimolecular fluorescence complementation, dimerization, phosphorylation

## Abstract

Signal transducer and activator of transcription 3 (STAT3) is a ubiquitous and pleiotropic transcription factor that plays essential roles in normal development, immunity, response to tissue damage and cancer. We have developed a Venus‐STAT3 bimolecular fluorescence complementation assay that allows the visualization and study of STAT3 dimerization and protein‐protein interactions in living cells. Inactivating mutations on residues susceptible to post‐translational modifications (PTMs) (K49R, K140R, K685R, Y705F and S727A) changed significantly the intracellular distribution of unstimulated STAT3 dimers when the dimers were formed by STAT3 molecules that carried different mutations (ie they were “asymmetric”). Some of these asymmetric dimers changed the proliferation rate of HeLa cells. Our results indicate that asymmetric PTMs on STAT3 dimers could constitute a new level of regulation of STAT3 signaling. We put forward these observations as a working hypothesis, since confirming the existence of asymmetric STAT3 homodimers in nature is extremely difficult, and our own experimental setup has technical limitations that we discuss. However, if our hypothesis is confirmed, its conceptual implications go far beyond STAT3, and could advance our understanding and control of signaling pathways.

**Abbreviations**
ATP: adenosine triphosphate
BiFC: bimolecular fluorescence complementation
BRET: bioluminescence resonance energy transfer
DIC: differential interference contrast
DelCT: deletion of the C‐terminus
EGFP: enhanced green fluorescent protein
FRET: fluorescence resonance energy transfer
LIF: leukemia inhibitory factor
PCR: polymerase chain reaction
PTMs: post‐translational modifications
SDS: sodium dodecyl sulfate
STAT3: signal transducer and activator of transcription‐3
V1: Venus 1 (amino acids 1‐158)
V2: Venus 2 (amino acids 159‐238)

## 1 INTRODUCTION

The signal transducer and activator of transcription 3 (STAT3) is a conserved transcription factor that plays key roles in development, immunity, response to injury and cancer.^1^, ^2^ STAT3 dimerization, post‐translational modification (PTM) and intracellular location are limiting events in these biological functions. STAT3 is most commonly found as homodimers in the cytosol of unstimulated cells, and is canonically activated by phosphorylation at Y705 upon stimulation with a variety of cytokines and growth factors.^1^, ^2^ Phosphorylated STAT3 is then retained in the nucleus, where it activates the transcription of a specific set of genes. However, unstimulated STAT3 is also found in the nucleus, binds to DNA and controls the transcription of a gene set different from phosphorylated STAT3, such as m‐Ras, RANTES or cyclin B1.^3^, ^4^, ^5^ Stimulation of cells with cytokines from the IL‐6 family or angiotensin II also induces accumulation of unphosphorylated STAT3 in the nucleus, where it forms complexes with other transcriptional regulators such as NFkB and p300/CBP.^6^, ^7^, ^8^ Nuclear accumulation of unphosphorylated STAT3 could have relevant physiopathological consequences, as it is correlated with cardiac hypertrophy and dysfunction in mice overexpressing Angiotensin receptor.^3^ Furthermore, de novo mutations that force nuclear accumulation of unphosphorylated STAT3, such as L78R, E166Q or Y640F, are associated with inflammatory hepatocellular adenomas.^9^, ^10^ STAT3 can be also found in the mitochondria, where it is necessary for normal activity of the electron transport chain.^11^ This function is independent of its activity as a transcription factor and Y705 phosphorylation, but dependent on S727 phosphorylation.^11^, ^12^ Mitochondrial STAT3 can also act as a transcription factor on mitochondrial DNA, and has been found to promote Ras‐mediated oncogenic transformation.^1^, ^13^ Other PTMs can regulate the behavior and function of STAT3, such as acetylation at K49 or K685 3, 14, 15 or dimethylation at K49 or K140.^16^, ^17^ Although dimethylation of the K49 or K140 residues is induced by stimulation with cytokines and is favored by STAT3 phosphorylation, there is basal K49 (but not K140) dimethylation in the STAT3 of unstimulated cells,^16^ and the same happens with STAT3 acetylation.^14^, ^15^ The role of these and other PTMs on mitochondrial functions of STAT3 remains unknown.

**Figure 1:**
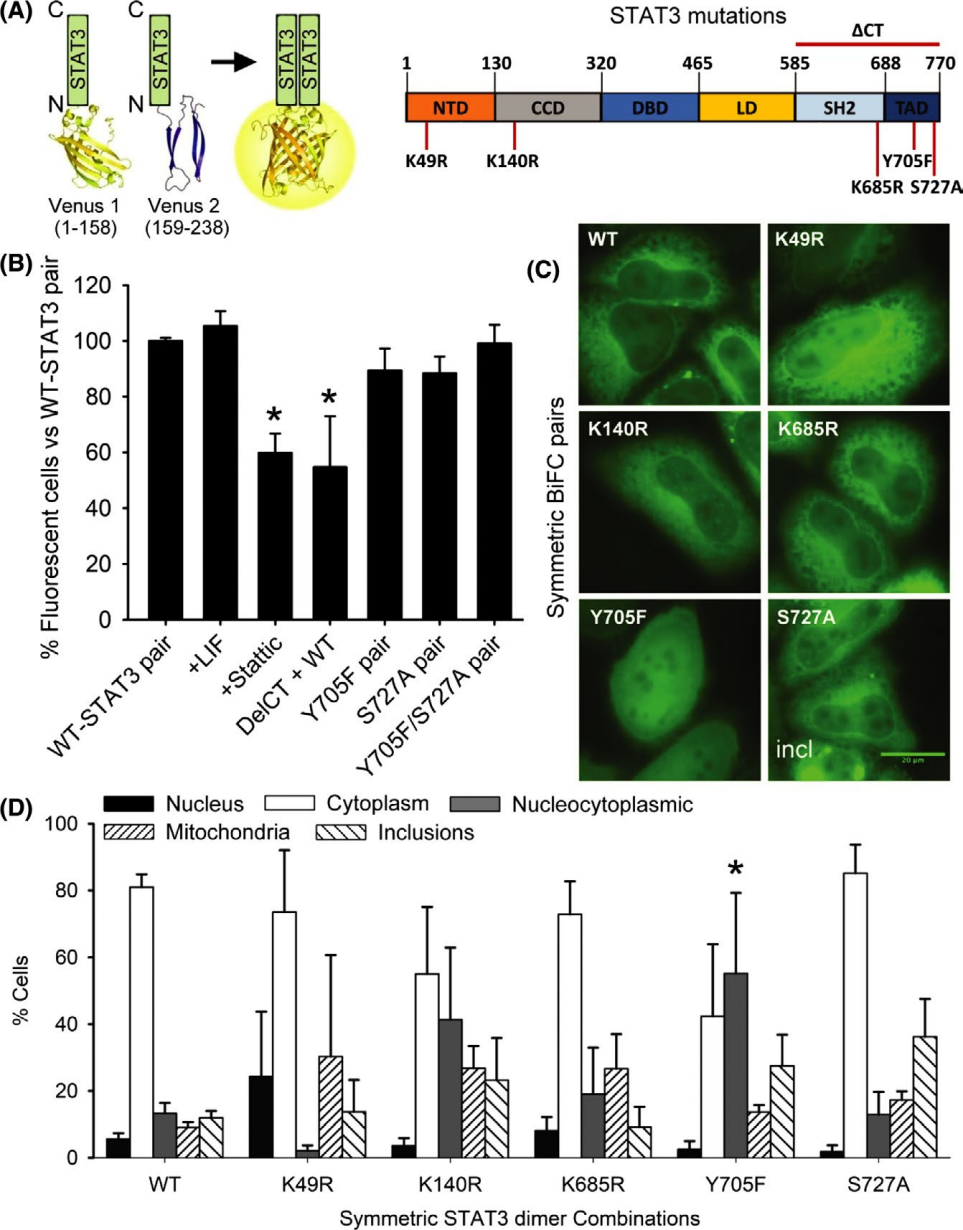
A Venus‐signal transducer and activator of transcription 3 (STAT3) bimolecular fluorescence complementation (BiFC) system allows the visualization and study of STAT3 homodimers in living cells. A, Venus BiFC fragments constituted by amino acids 1‐158 (Venus 1, V1) and 159‐238 (Venus 2, V2) were fused to the N‐terminus of the STAT3 sequence in two independent constructs. K49, K140, K685, Y705 and S727 residues can be post‐translationally modified, and were inactivated in both V1‐ and V2‐STAT3 constructs by site‐directed mutagenesis. B, Wild‐type (WT) Venus‐STAT3 constructs produced fluorescence in HeLa cells, and it was monitored by flow cytometry 24 h after transfection with BiFC constructs. Incubation with leukemia inhibitory factor (100 ng/mL) for 2 h in the absence of serum or the presence of the indicated drugs or mutant BiFC pairs (n = 3; *P* < .05). Results were normalized vs the WT STAT3 pair (100%). C, Microscopy pictures of representative cell phenotypes in the different symmetric combinations of BiFC Venus‐STAT3 constructs (Incl, inclusions; scale bar, 20 µm). D, Percentage of cells displaying fluorescence predominantly in the Nucleus (black bar), predominantly in the Cytosol (white bar), homogeneously distributed in cytoplasm and nucleus (nucleocytoplasmic, grey bar), in the mitochondria or in non‐mitochondrial inclusions. Data are shown as the average ± SEM of n = 12 WT or n = 3 (rest of combinations) independent experiments. *Sign. vs the symmetric WT STAT3 pair, *P* < .05

Three ingenious systems have been developed so far to visualize and study STAT3 dimerization in living cells, based on fluorescence resonance energy transfer (FRET),^18^ bioluminescence resonance energy transfer (BRET) 5 or the homoFluoppi tag.^19^ The FRET/BRET systems enable the visualization of both STAT3 homodimerization and its interaction with other proteins in real time and in a reversible manner.^5^, ^18^ However, they require very skilled users for sampling and analyses and are difficult to adapt for high‐throughput experiments. The homoFluoppi system is simpler but it only allows to visualize STAT3 homodimerization, and exclusively by microscopy, as there is no change in total fluorescence but in the distribution of the fluorescence within the cell, in the form of punctae.^19^ Bimolecular Fluorescence complementation (BiFC) assays also allow the analysis of protein‐protein interactions in living cells,^20^ and their particular properties make them complementary to FRET/BRET or homoFluoppi systems.^20^, ^21^ In BiFC assays, the proteins of interest are fused to two non‐fluorescent, complementary fragments of a fluorescent reporter, such as Venus (Figure 1A). When the proteins of interest dimerize, the fragments are brought together and reconstitute the fluorophore, the fluorescence being proportional to the amount of dimers. This fluorescence can be easily recorded and quantified by microscopy or flow cytometry in living cells, and applied to high‐throughput setups.

Here, we developed a suit of Venus‐STAT3 BiFC constructs that are not only an important addition to the existing STAT3 toolset, but were also successfully employed to generate an interesting hypothesis on the control of the STAT3 pathway by PTMs. Literature on STAT3 generally assumes that STAT3 homodimers are formed by two identically modified molecules. However, this is highly unlikely in a complex intracellular context, as PTMs do not occur in all the pool of STAT3 molecules at the same time or with the same efficiency. We aimed at determining the relative contribution of residues K49, K140, K685, Y705 and S727 to the dimerization and intracellular distribution of STAT3 homodimers.

## 2 RESULTS

### 2.1 Development and validation of a Venus‐STAT3 system

We developed a suit of plasmids to study STAT3 dimerization in living cells, based on BiFC systems using Venus fragments as a reporter (Figure 1A), as we did for other proteins in previous reports.^20^, ^22^, ^23^ When STAT3 dimerizes, the Venus fragments are brought together and reconstitute the fluorophore, fluorescence being proportional to the amount of dimers (Figure S1A). Transfection of HEK293 or HeLa cells with the wild‐type (WT) pair of Venus‐STAT3 BiFC constructs led to successful expression of the chimeric proteins V1‐STAT3 and V2‐STAT3 (Figure 1B,C; Figure S1A). Fluorescence was primarily cytoplasmic in both cell lines, with low but visible nuclear signal (Figure 1C; Figure S1B). The combination of STAT3 with the corresponding BiFC constructs for Mdm2 or p53 proteins had extremely low levels of fluorescence (Figure S2A). This is consistent with the fact that these proteins are not STAT3 interactors and supports the specificity of the Venus‐STAT3 BiFC system.

Incubation with leukemia inhibitory factor (LIF, 100 ng/mL) induced STAT3 phosphorylation and translocation to the nucleus in HEK293 and HeLa cells (Figure S1B,C), but it did not enhance STAT3 dimerization (Figure 1B; Figure S1D). Incubation with STAT3 inhibitor Stattic (5 µmol/L) or removal of the C‐terminus containing the SH2 domain partially prevented STAT3 dimerization (Figure 1B), consistent with previous reports.^18^, ^24^ On the other hand, single or double Y705F/S727A phosphoresistant mutants did not decrease fluorescence (Figure 1B). These results support existing evidence indicating that STAT3 dimerization is actually independent of phosphorylation.^5^, ^19^, ^25^

Naturally occurring STAT3 mutations cause hyper‐immunoglobulin E syndrome or inflammatory hepatocellular adenoma.^10^, ^26^ The L78R mutation, in particular, inhibits STAT3 dimerization but has a strong tendency to go to the nucleus and activate transcription.^10^, ^18^ We created a L78R STAT3 mutant in our BiFC system and confirmed first that it inhibited STAT3 dimerization (Figure S2A), and induced nuclear translocation at the expense of cytoplasmic STAT3 (Figure S2B,C). Furthermore, the analysis of microscopy images indicated that it also induces STAT3 aggregation into cytoplasmic inclusions (Figure S2B,C).

Taken altogether, our results indicate that the behavior of the Venus‐STAT3 BiFC system is consistent with previous reports for tagged STAT3, and indicates that it could be useful for the analysis of environmental or genetic modifiers of STAT3 dimerization, protein‐protein interactions and intracellular traffic.

### 2.2 The dimerization and intracellular distribution of unstimulated symmetric STAT3 homodimers

Next, we tried to elucidate the role that particular residues susceptible to PTMs could play on the dimerization and intracellular localization of STAT3 homodimers without adding exogenous cytokines. The residues chosen were K49, K140, K685, Y705 and S727, susceptible to acetylation, methylation or phosphorylation. The original idea was to establish a baseline for future experiments in the presence of cytokines, which enhance the frequency of these particular PTMs in STAT3. However, low levels of these PTMs in the absence of cytokines have been also described in the literature,^3^, ^14^, ^15^ and we also wanted to know if these basal PTMs or the residues themselves had any influence in the dimerization and distribution of STAT3 homodimers. We initially assumed that the two STAT3 molecules that form a dimer are identical in all aspects, including their PTMs. Therefore, our analyses focused first on “symmetric” combinations. No combination had a consistent effect on unstimulated STAT3 dimerization, as determined by flow cytometry (Figure S3). In order to analyze the intracellular location of unstimulated STAT3 homodimers, we classified cells qualitatively in three categories that are mutually exclusive (their sum is 100% of cells), according to the relative intensity and location of the fluorescence signal (Figure 1C,D; Figure S2): (a) predominantly in the cytoplasm (eg WT pair); (b) predominantly in the nucleus (eg upon LIF induction, Figures S1B and S2B); or (c) homogeneously distributed through nucleus and cytoplasm (eg Y705F pair). We also determined the percentage of cells with mitochondrial signal or intracellular inclusions (Figure S4). Although changes in patterns of STAT3 dimer distribution were observed in several symmetric BiFC pairs, only the Y705F pair induced a significant increase in the percentage of cells with homogeneous nucleocytoplasmic fluorescence (Figure 1D).

### 2.3 Relative contribution of specific residues to STAT3 dimerization, intracellular location, and cell proliferation

**Figure 2:**
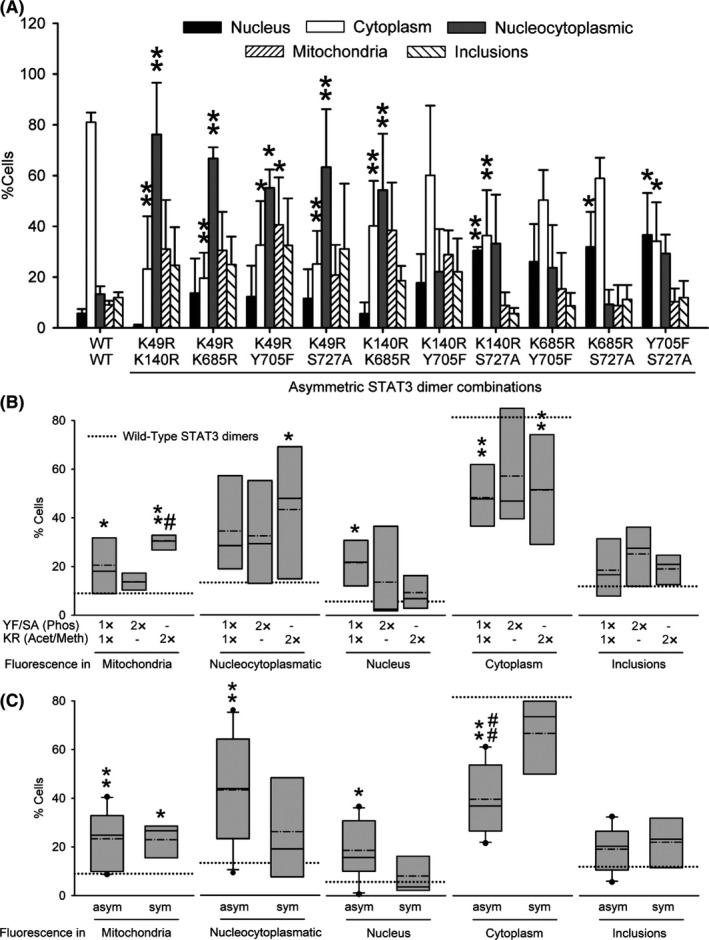
Asymmetric signal transducer and activator of transcription 3 (STAT3) post‐translational modifications regulate intracellular distribution of STAT3 homodimers. A, Intracellular distribution of fluorescence in asymmetric combinations of Venus‐STAT3 bimolecular fluorescence complementation (BiFC) constructs (and the WT symmetric pair as reference). Data are shown as the average of n = 12 (WT, wild‐type) or n = 3 (rest of combinations) independent experiments ± SEM. Statistical analysis was carried out by means of a one‐way ANOVA followed by a Bonferroni test adjusted for multiple comparisons. Significant vs the symmetric WT STAT3 pair, *P < .05, **P < .01. B and C, The same original data, but pooled according to the number and nature of substitutions (B) or the symmetry or asymmetry of substitutions (C) in the STAT3 homodimer, and represented as box plots. The limits of the boxes represent the smallest and largest values, the straight line represents the median, the dashed line represents the average, and the dotted line represents the average for WT STAT3 pair. Statistical analysis was carried out on the Average ± SEM of each pool of data (1×YF/SA:1×KR, n = 6; 2×YF/SA, n = 3; 2×KR, n = 6; sym, n = 5; asym, n = 10). Significant vs the symmetric WT STAT3 pair, *P < .05, **P < .01; significant vs 2×YF/SA substitution (B) or the symmetric mutant pairs (C), ^#^ *P* < .05, ^##^ *P* < .01

Like us, and to the best of our knowledge, the existing scientific literature on STAT3 implicitly assumes that STAT3 homodimers are formed by two identical molecules in all aspects, including PTMs. For example, it is still relatively easy to find articles and reviews where STAT3 is described to homodimerize only upon phosphorylation of both molecules at Y705,^27^ and ourselves worked under this same assumption until very recently.^28^ Here, we made use of the unique properties of our BiFC system to determine the relative contribution of each residue to the dimerization and intracellular distribution of unstimulated STAT3 dimers, in an experimental paradigm similar to the one we used previously for mutant huntingtin.^20^ We combined all possible inactivating PTM mutations with each other, but again no combination had a consistent effect on unstimulated STAT3 dimerization (Figure S3). However, the intracellular distribution of STAT3 homodimers was significantly altered by specific combinations of STAT3 molecules (Figure 2A). Unlike the K49R symmetric pair, K49R asymmetric combinations dominantly induced an increase in cells with homogeneous nucleocytoplasmic fluorescence at the expense of cytoplasmic location (Figure 2A), similar to the Y705F symmetric pair. K140R‐ or K685R‐containing pairs showed some tendency to shift cytoplasmic location toward nucleus, but only the K140R + S727A combination achieved significance. This phenotype was almost identical to the Y705F + S727A asymmetric pair (Figure 2A).

We then pooled and analyzed all results according to the number and type of PTM mutations present in each BiFC pair. Combinations carrying any one (asymmetric) or two K‐R substitutions (symmetric or asymmetric) significantly increased mitochondrial translocation, while decreasing the percentage of cells with STAT3 dimers predominantly in the cytoplasm (Figure 2B). Asymmetric combinations of one K‐R substitution and one phosphoresistant mutant also increased nuclear translocation, but only 2×K‐R combinations increased homogeneous nucleocytoplasmic distribution. Combinations carrying any two phosphoresistant mutations (symmetric or asymmetric) had no significant effect on cellular distribution of STAT3 homodimers (Figure 2B). These results indicate that specific asymmetric PTMs on STAT3 dimers can prevent their nuclear import/export. This was later confirmed by pooling the data according to whether the STAT3 pair was symmetric or asymmetric in their PTM mutations (Figure 2C). We found that only asymmetric PTM mutant combinations increased nucleocytoplasmic or nuclear distribution at the expense of decreasing cytoplasmic localization of STAT3 homodimers. Asymmetric combinations were also sufficient to produce an increase in mitochondrial localization of STAT3 dimers (Figure 2C).

**Figure 3:**
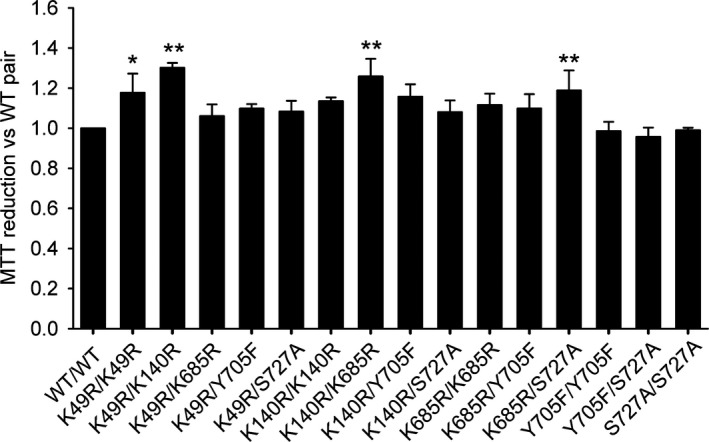
Specific asymmetric signal transducer and activator of transcription 3 (STAT3) dimers enhance the proliferation of HeLa cells. HeLa cells were transfected with the different combinations of STAT3 bimolecular fluorescence complementation constructs, and their viability was determined by means of the 3‐(4,5‐dimethyl‐2‐thiazolyl) 2,5‐diphenyl‐2H‐tetrazolium bromide assay 24 h later. Statistical analysis was carried out on the Average ± SD of data (n = 3). Significant vs the symmetric wild‐type (WT) STAT3 pair, *P < .05, **P < .01

Signal transducer and activator of transcription 3 contributes to cancer cell survival, proliferation and malignant transformation even in conditions where it is not stimulated by cytokines,^25^, ^29^, ^30^, ^31^, ^32^ and mitochondrial STAT3 could promote oncogenic transformation in certain biological contexts.^13^, ^33^ In order to know if there were biological consequences of the observed changes of behavior in unstimulated STAT3 dimers, HeLa cells were transfected with the different combinations of constructs and their proliferation was determined 24 hours later (Figure 3). The asymmetric combinations K49R/K140R, K140R/K685R and K685R/S727A increased significantly the number of cells versus control cultures transfected with wild type STAT3. Among the symmetric combinations, only the K49R pair showed a smaller but significant increase in cell proliferation. These results indicate that asymmetric dimers of STAT3 could have differential biological effects.

## 3 DISCUSSION

We have developed and validated a new BiFC assay for the visualization and study of STAT3 interactions in living cells. Our system responds as expected to pharmacological activation or inhibition of STAT3, disease‐associated genetic mutations, and potential protein interactors. The Venus‐STAT3 BiFC system is complementary to previously reported FRET,^18^ BRET^5^ or homofluoppi,^19^ as they all have different advantages and limitations. FRET/BRET approaches enable the visualization of any protein‐protein interaction and have high temporal and spatial resolution, but they are difficult to scale‐up to high‐content screenings. Homofluoppi enables high‐throughput analysis, but it is not suitable for the visualization of STAT3 heterodimers (eg with STAT1) or other protein‐protein interactions. Both are especially suitable for microscopy analyses, but not for flow cytometry analyses. BiFC systems can be applied to any type of protein‐protein interaction, are easy to use and scale up for high‐throughput analysis, and enable both microscopy and flow cytometry approaches. However, BiFC systems have a lower time resolution than FRET, BRET or Homofluoppi systems and usually lower signal‐to‐noise ratios.^34^ The reconstitution of the fluorophore is irreversible, potentially limiting interactions that are transient, but otherwise having the advantage of accumulating low frequency events that otherwise would be unnoticed. And finally, only dimers with complementary reporter fragments will be observed (ie Venus 1 + Venus 2), but it is possible that dimers are also formed between STAT3 molecules that carry the same Venus fragment. Nevertheless, BiFC systems are widely used,^34^ represent an excellent first, simple approach to visualize protein‐protein interactions in living cells,^35^ and could even be combined with FRET approaches for the visualization of multi‐protein complexes.^36^ We believe that our STAT3 BiFC system will make a great addition to the existing STAT3 protein‐protein interaction toolbox.

Our results indicate that asymmetric PTMs could constitute a new level of regulation of unstimulated STAT3 behavior and function. We must emphasize that this observation was very surprising and is put forward cautiously as a working hypothesis, rather than a conclusive result. To the best of our knowledge, there is no direct empirical evidence in the literature showing that asymmetrically modified STAT3 dimers actually happen in nature, and such demonstration would be currently extremely difficult from a technical point of view, even in vitro. Previous studies most often rely on systems that do not differentiate between monomers and dimers,^3^, ^4^, ^11^, ^12^, ^14^, ^15^, ^17^, ^37^ and/or that produce a single population of STAT3 molecules, either mutated or normal.^5^, ^18^, ^19^ And yet, in the crowded and diverse intracellular environment, the probability for two identical STAT3 molecules to form a dimer (or for a dimer to be modified in both molecules simultaneously and in the same residues) should be low, although it could certainly be enhanced by either total absence or presence of stimuli. For example, most STAT3 molecules are not phosphorylated in the absence of extracellular stimuli, and this proportion is reversed shortly after cytokines bind to their corresponding membrane receptors (Figure S1C). However, cells often show small amounts of phosphorylated STAT3 in resting state (Figure S1C) and, conversely, cytokine‐stimulated STAT3 induces the de novo transcription of new STAT3 molecules that are not necessarily phosphorylated.^1^, ^2^ This indicates that unphosphorylated and phosphorylated STAT3 should coexist at similar levels in many situations, and the literature presents evidence that this could be equally true for other STAT3 PTMs induced by cytokines.^14^, ^15^, ^16^, ^17^

Beyond the technical difficulties to confirm the existence of asymmetric STAT3 homodimers in nature, our experimental design has several limitations that may have determined our observations. First, we have tested our system in cells that express endogenous STAT3, which could somewhat interfere with the system. One argument against this possibility is that we observe changes in asymmetric combinations but not in symmetric combinations. We initially assumed endogenous STAT3 would interfere homogeneously in all possible combinations. If this is incorrect and endogenous STAT3 is interfering, especially with certain combinations, we would expect some degree of similarity between symmetric and asymmetric combinations having at least one mutation in common. However, symmetric combinations are similar between them and in most cases different to their asymmetric counterparts. It should also be noted that, while endogenous and exogenous STAT3 could carry different PTMs (besides the BiFC tags), a possible differential interference of endogenous STAT3 does not necessarily invalidate our hypothesis. In normal conditions, the pool of STAT3 molecules will be heterogeneous, and the possible differential interference of endogenous STAT3 could actually correspond to the effect that other molecules would have on specific STAT3 homodimers. Nevertheless, the experiments should certainly be repeated in a STAT3 knockout context to remove possible confounders.

Second, we overexpressed the constructs transiently, probably contributing to the high variability we observe between experiments. Higher‐than‐normal levels of STAT3 could produce interactions that would not occur in normal conditions. It was suggested to us that stable transfection on STAT3‐negative cells could both improve variability and produce levels of STAT3 similar to the endogenous levels in a parental cell line. This is not necessarily correct, since expression of proteins highly depend on their promoter, and similar attempts in the literature produced cell lines expressing higher levels than parental cell lines.^16^ Although stable transfection or infection with viruses could be pursued in the future, this approach could also be problematic because of the particular features of BiFC systems, such as its irreversibility, which could produce further accumulation of STAT3 dimers over time.

Third, BiFC assays have their own technical limitations. BiFC assays frequently show some spontaneous reconstitution of the fluorophore that adds background and reduces the signal‐to‐noise ratio.^34^ We present results that indicate that our system is specific, combining STAT3 with proteins that should not interact with it or introducing pharmacological or genetic modifiers of STAT3 dimerization (Figure 1B; Figure S2). However, we never achieved total inhibition of STAT3 homodimerization, and therefore some possible background cannot be completely ruled out. Such possible background could produce artifacts, making us believe that we are visualizing actual STAT3 dimers when we are just observing reconstituted Venus, and in this situation STAT3 monomers could behave differently. Furthermore, the irreversibility of the BiFC systems could amplify the occurrence of low frequency interactions, therefore magnifying events that are not biologically relevant. These two last issues could be overcome by confirming our observations in a FRET system, alone or in combination with our BiFC system.^36^ Alternatively, STAT3 mutants could be inserted in existing split luciferase systems that are reversible and have higher signal‐to‐noise ratio than BiFC assays.^38^

In summary, our results must be considered as a working hypothesis, but they point at an exciting possibility: the behavior and function of protein homodimers could be controlled by PTMs in only one of the molecules. If asymmetric STAT3 dimers actually happen and play a relevant biological role, this would open a series of interesting questions: do they regulate specific sets of genes? Do they enable gradation of STAT3 transcriptional or mitochondrial activities? And if they do not happen, how do cells manage to achieve perfectly symmetrical STAT3 dimers with such high efficiency? Given the essential roles of STAT3 in development, immunity, tissue stress and cancer, addressing these questions could have important implications for the diagnosis, treatment and understanding of a wide spectrum of human pathologies.

## 4 MATERIALS AND METHODS

### 4.1 Reagents

HeLa human cervix adenocarcinoma cells and HEK293 human embryonic kidney cells were acquired from ATCC (references CRM‐CCL‐2 and CRL‐1573, respectively), LIF from R&D systems, and Stattic from Selleckchem.

### 4.2 Cell cultures

HeLa and HEK293 cells were maintained in Dulbecco's minimal essential medium (DMEM; Gibco, Invitrogen) supplemented with 10% fetal bovine serum (FBS) and 1% of a penicillin/streptomycin commercial antibiotic mixture (Gibco; Invitrogen), under controlled conditions of temperature and CO_2_ (37°C, 5% CO_2_). Cell culture dishes were purchased from Techno Plastic Cultures (AG) unless otherwise indicated. For all experiments, cells were seeded at a density of 10 000 cells/cm^2^ regardless dish size. For flow cytometry assays, cells were grown on 6‐well plates (35 mm diameter). For cell viability and adenosine triphosphate (ATP) assays, cells were grown on 96‐well and 24‐well dishes, respectively. For microscopy, cells were seeded on glass‐bottom 35 mm dishes (10 mm glass surface diameter; IBIDI) and fixed with 4% paraformaldehyde in phosphate buffered saline (PBS) right before imaging. For protein extraction (PAGE and filter trap assays) cells were seeded on 60 or 100 mm dishes.

### 4.3 Plasmids

Venus‐STAT3 BiFC constructs were designed using A Plasmid Editor free online software (http://jorgensen.biology.utah.edu/wayned/ape/) and synthesized by Invitrogen. Briefly, the cDNA sequence of STAT3‐alpha was fused to the sequence of two complementary, non‐fluorescent fragments of the Venus protein (Venus 1, amino acids 1‐157; and Venus 2, amino acids 158‐238) (Figure 1A), and inserted in a pcDNA 3.3 TOPO backbone (Invitrogen). Mutant constructs were produced by polymerase chain reaction (PCR)‐based site‐directed mutagenesis using these original constructs as templates. Table 1 shows the primers used for cloning and mutagenesis. All BiFC constructs were deposited in Addgene (https://www.addgene.org/). Deletion mutants lacking the C‐terminus (DelCT) of STAT3 were produced by PCR‐mediated subcloning using full‐length Venus‐STAT3 BiFC constructs as templates. The original lysine (K) residues on positions 49, 140 and 685 were replaced by arginine (R) residues, the tyrosine (Y) residue on position 705 by phenylalanine (F) and the serine (S) residue on position 727 by alanine (A) (Figure 1A). Additionally, the L78R mutation associated to inflammatory hepatocellular adenoma was also produced and analyzed during the optimization of the system. Plasmid transfection was carried out by means of JetPrime (Polyplus‐transfection) following manufacturer's instructions. Subsequent cell viability, ATP, immunoblotting, microscopy and flow cytometry assays were carried out 24 hours after transfection.

**Table 1:** Primers for mutagenesis or polymerase chain reaction (PCR) cloning

STAT3 K49R	Fwd	CCCCTTGGATTGGGAGTCAAGATTG
	Rev	CAATCTTGACTCCCAATCCAAGGGG
STAT3 K140R	Fwd	GGTGACGGAGACACAGCAGATGCTG
	Rev	CAGCATCTGCTGTCTCTCCGTCACC
STAT3 K685R	Fwd	GAGGCATTCGGAAGGTATTGTCGGCC
	Rev	GGCCGACAATACCTTCCGAATGCCTC
STAT3 Y705F	Fwd	CAGGTAGCGCTGCCCCATTCCTGAAGACCAAGTTTATC
	Rev	GATAAACTTGGTCTTCAGGAATGGGGCAGCGCTACCTG
STAT3 S727A	Fwd	CATTGACCTGCCGATGGCACCCCGCACTTTAGATTC
	Rev	GAATCTAAAGTGCGGGGTGCCATCGGCAGGTCAATG
V1‐STAT3 ΔCT	Fwd	ACTAGCTAGCATGGTGAGCAAGGGCGAGGA
	Rev	CTATGGATCCTTAGTTCCAAAGGGCCAGGA
STAT3 L78R	Fwd	CAAGAGTCGAATGTTCGCTATCAGCACAATCTAC
	Rev	GTAGATTGTGCTGATAGCGAACATTCGACTCTTG

### 4.4 Flow cytometry

Cells were washed once with PBS (Gibco, Invitrogen), trypsinized (0.05% w/v, 37°C, 5 minutes) and collected into BD Falcon Round‐Bottom Tubes (BD Biosciences). Cells were then resuspended in PBS at room temperature and analyzed by means of a Calibur flow cytometer (Beckton Dickinson). Ten thousand cells were analyzed per experimental group. The FlowJo software (Tree Star Inc) was used for data analysis and representation.

### 4.5 Microscopy

Images of transfected HeLa or HEK293 cells were acquired using an Applied Precision DeltavisionCORE system, mounted on an Olympus inverted microscope, equiped with a Cascade II 2014 EM‐CCD camera, using a 63× 1.4NA Oil immersion objetive, DAPI + DsRed + enhanced green fluorescent protein (EGFP) fluorescence filtersets and differential interference contrast (DIC) optics. Pictures were analyzed by means of the ImageJ free online software (http://rsbweb.nih.gov/ij/).

### 4.6 Western blotting

Twenty‐four hours after transfection, cells were washed once with PBS, lysed in a triton‐based lysis buffer (1% NP‐40, 150 mmol/L NaCl, 50 mmol/L Tris pH 7.4, supplemented with protease inhibitor and phosphatase inhibitor cocktails [Roche diagnostics]). Lysates were sonicated for 10 seconds at 5 mA using a Soniprep 150 sonicator (Albra) and centrifuged at 10 000× g for 10 minutes at 4°C, and supernatants were collected for analyses. Protein concentration was quantified by means of the Bradford assay. Equal amounts of protein (30‐50 μg) from each extract were prepared for analysis by western blotting under denaturing conditions. Loading buffer (200 mmol/L Tris‐HCl pH 6.8; 8% sodium dodecyl sulfate (SDS); 40% glycerol; 6.3% β‐mercaptoethanol; 0.4% bromophenol blue) was added to the samples, which were then boiled for 5 minutes at 95°C and resolved on 12% SDS‐polyacrylamide gel electrophoresis with SDS‐containing running. Proteins were then transferred to nitrocellulose membranes, and transfer efficiency and equal sample loading was confirmed by Ponceau S staining. Membranes were blocked with 5% non‐fat dry milk in Tris‐HCl buffer saline‐Tween (TBS‐T) (150 mmol/L NaCl, 50 mmol/L Tris pH 7.4, 0.5% Tween‐20) for 1 hour at room temperature before addition of primary antibodies. Primary antibodies against the following proteins were used at the specified concentrations: STAT3 (1:1000; Cell Signaling), phospho‐STAT3 (Tyr705) (1:1000; Cell Signaling); and GAPDH (1:30 000; Ambion). Membranes were then washed 3 times in TBS‐T and incubated with a secondary mouse IgG Horseradish Peroxidase‐linked antibody (1:10 000; GE Healthcare Life Sciences). Signals were developed by enhanced chemiluminescence reagents (Millipore) and imaged with a Chemidoc device (Biorad).

### 4.7 Cell proliferation assay

Twenty‐four hours after transfection, 10 μL of 3‐(4,5‐dimethyl‐2‐thiazolyl) 2,5‐diphenyl‐2H‐tetrazolium bromide (MTT) solution in PBS (2 mg/mL) was added to each well, and incubated for 2 hours at 37°C and 5% CO_2_. Then, medium was discarded and 100 μL of 100% dimethyl sulfoxide were added. Cells were lysed and MTT precipitates solubilized for 15 minutes at room temperature, and absorbance was measured in an automatic microplate reader (Sunrise 8708; Tecan Trading AG) at 570 nm.

### 4.8 Statistics

Sigmaplot software (Systat Software, Inc) were used to perform the statistical analysis and graphical representation of data. Results are shown as the average ± SEM of at least 3 independent experiments, as indicated in figure legends. Statistical analysis was carried out by means of a one‐way ANOVA followed by a Bonferroni test adjusted for multiple comparisons. Results were in all cases considered significant only when *P* < .05.

## CONFLICT OF INTEREST

The authors declare no conflicts of interest.

## AUTHOR CONTRIBUTIONS

RV is responsible for cloning, mutagenesis and flow cytometry studies, cell viability assays and part of microscopy analyses, and BC is responsible for most microscopy studies. CSA and AMR are responsible for the first optimization experiments with the BiFC system and LIF experiments, a small part of which are shown in Figure S1. FM and JBS contributed to the microscopy, flow cytometry and immunoblotting at different stages of the project. CR and VM contributed to the analysis, presentation and interpretation of data. MSM contributed to cloning and mutagenesis of STAT3 BiFC constructs. FH had the original idea, designed the experiments, arranged the final figures and analyzed the data, coordinated the project and wrote the manuscript.
