# Supplementary figures and images for: Can asymmetric post‐translational modifications regulate the behavior of STAT3 homodimers?
Source: FASEB Bioadv. 2020 Jan 27;2(2):116–25. doi: 10.1096/fba.2019-00049 (PMC7003655; doi:10.1096/fba.2019-00049)

Supplementary Figure 1

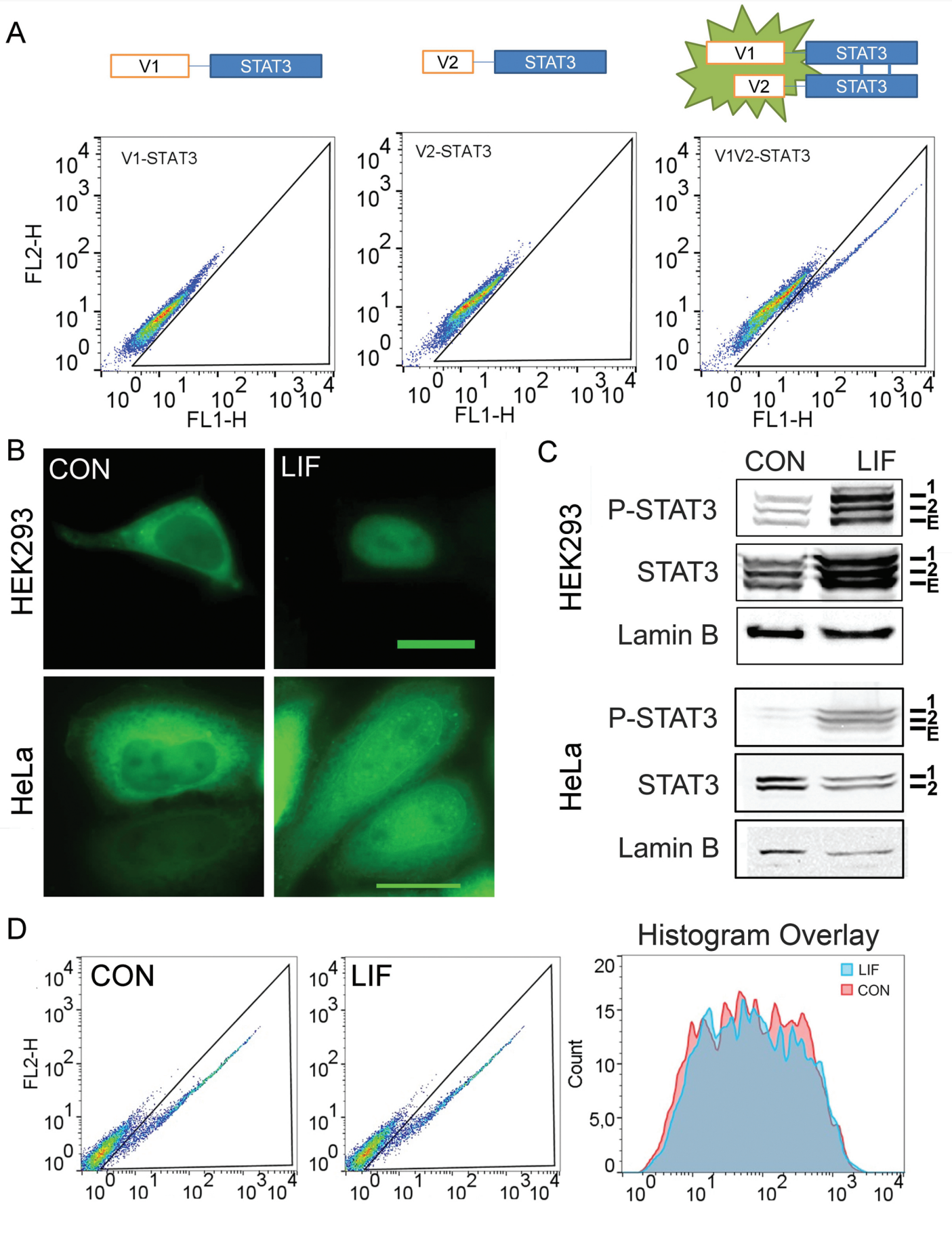

Supplement: Supplementary file 1 [file FBA2-2-116-s001.pdf]

Supplementary Figure 2

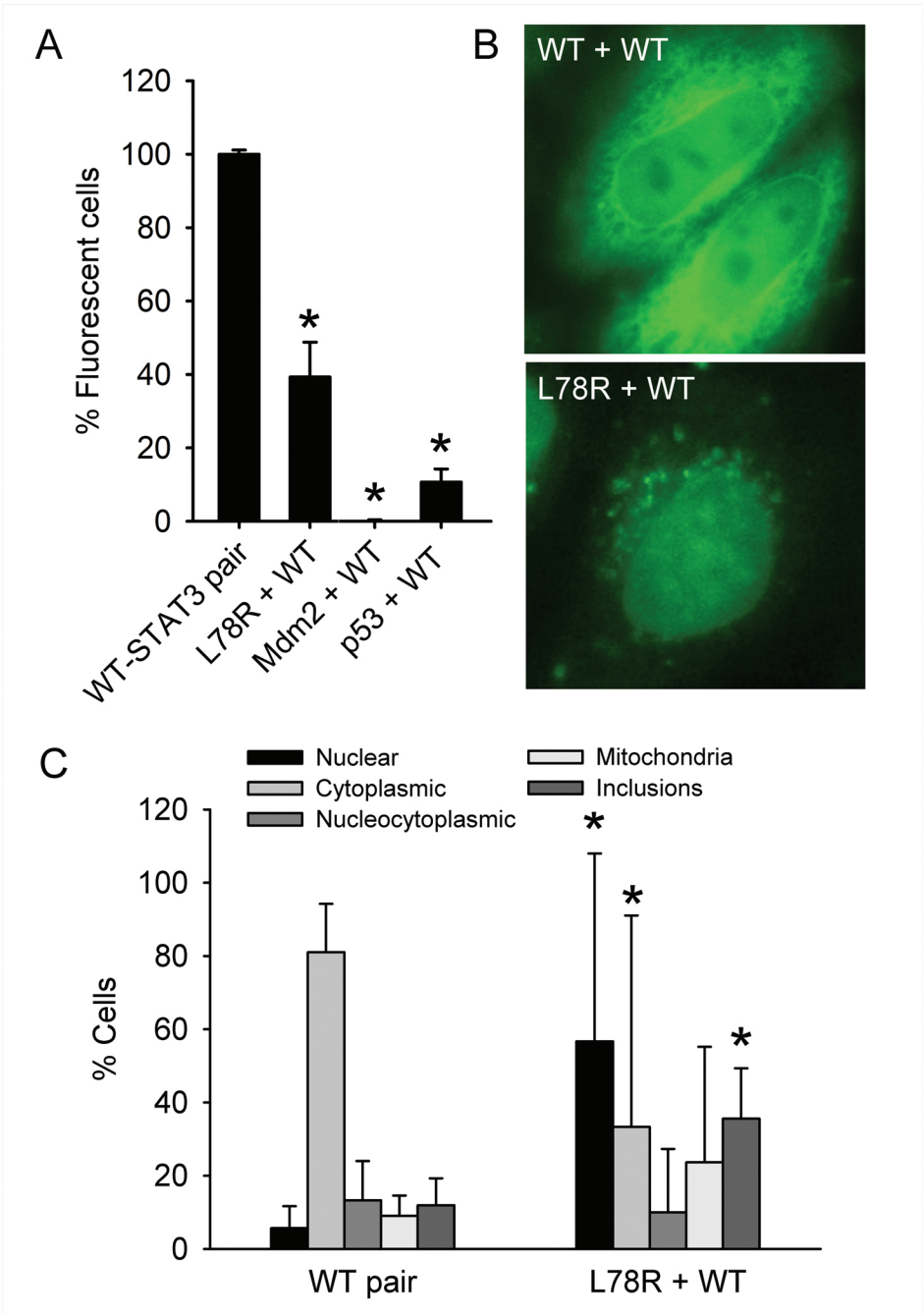

Supplement: Supplementary file 2 [file FBA2-2-116-s002.pdf]

Supplementary Figure 3

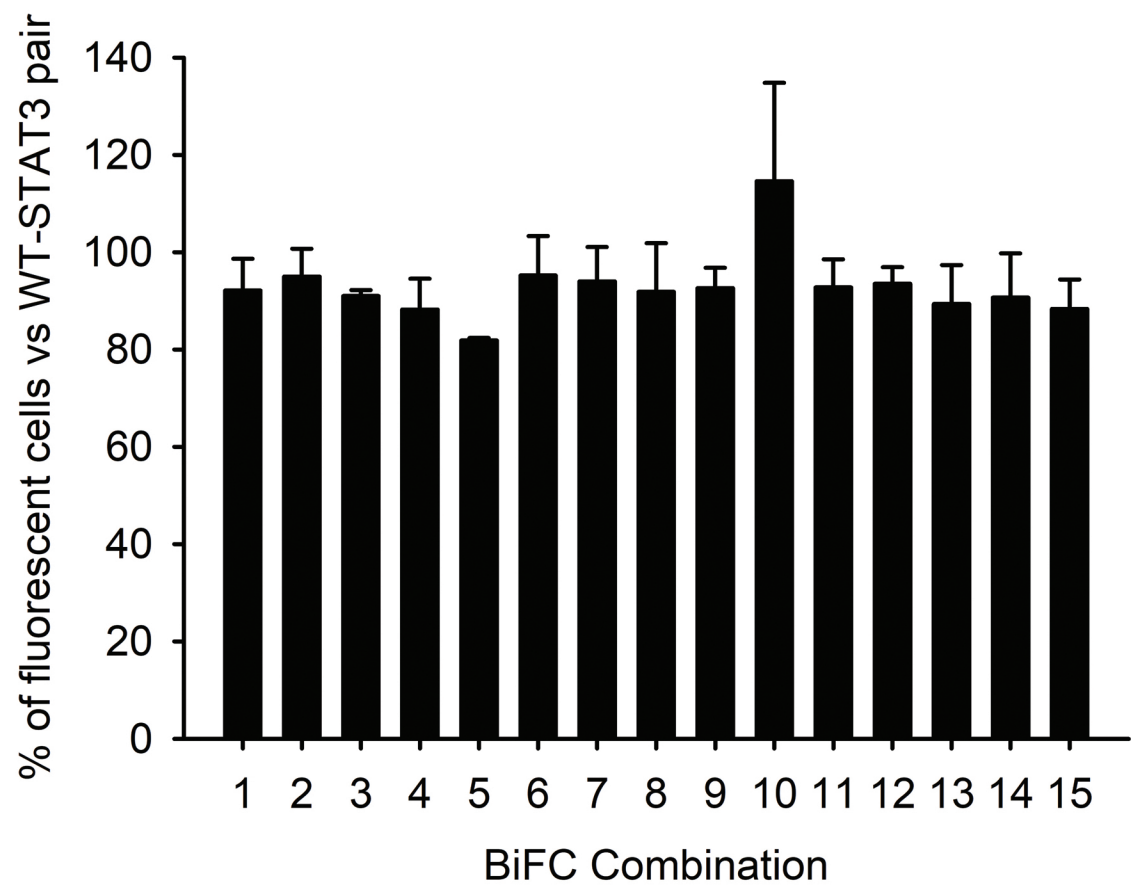

|       | K49R | K140R | K685R | Y705F | S727A |
|-------|------|-------|-------|-------|-------|
| K49R  | 1    |       |       |       |       |
| K140R | 2    | 6     |       |       |       |
| K685R | 3    | 7     | 10    |       |       |
| Y705F | 4    | 8     | 11    | 13    |       |
| S727A | 5    | 9     | 12    | 14    | 15    |

Supplement: Supplementary file 3 [file FBA2-2-116-s003.pdf]

Supplementary Figure 4

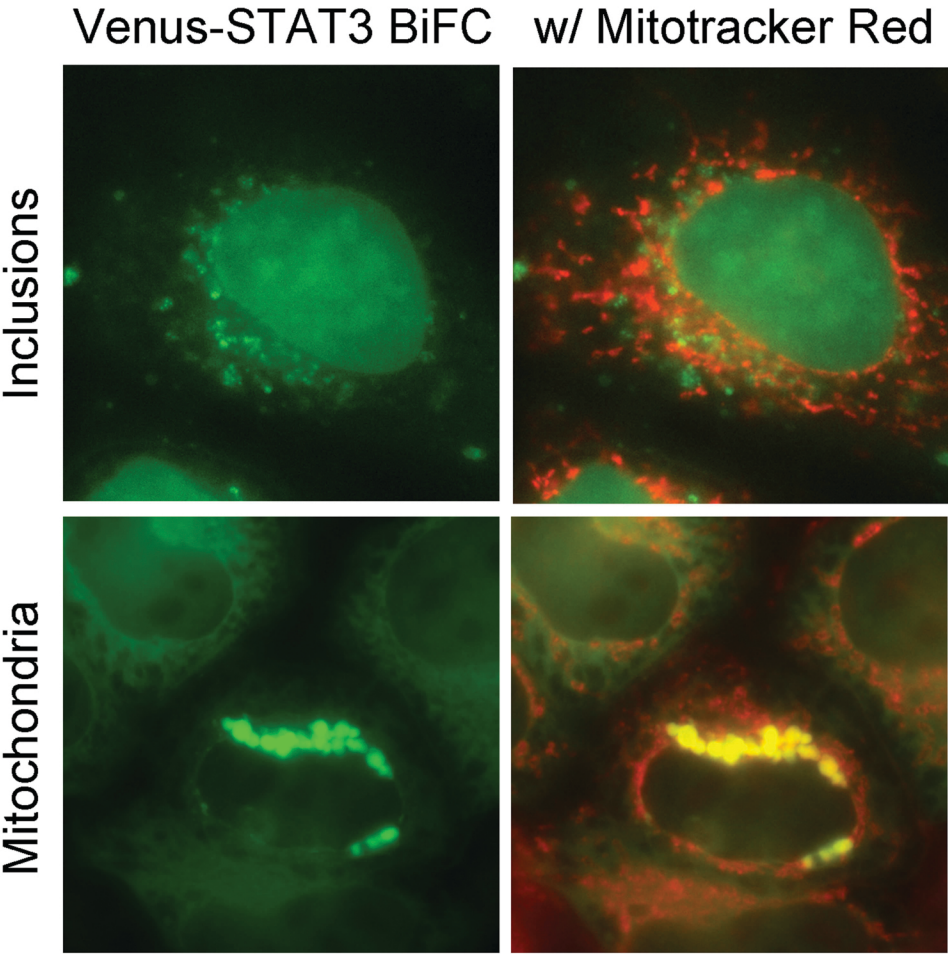

Supplement: Supplementary file 4 [file FBA2-2-116-s004.pdf]
