# Supplementary material for: Can asymmetric post‐translational modifications regulate the behavior of STAT3 homodimers?
Source: FASEB Bioadv. 2020 Jan 27;2(2):116–25. doi: 10.1096/fba.2019-00049 (PMC7003655; doi:10.1096/fba.2019-00049)
Supplement: Supplementary file 5 [file FBA2-2-116-s005.docx]

**Figure S1.** The Venus-STAT3 BiFC system allows to monitor nuclear translocation of STAT3 dimers upon stimulation with LIF. A, Flow cytometry charts showing that V1-STAT3 and V2-STAT3 BiFC constructs do not produce fluorescence by themselves, but they fluoresce when they are transfected together. B, Representative fluorescence microscopy pictures of HEK293 or HeLa cells transfected with Venus-STAT3 constructs and incubated in the presence or absence of Leukemia Inhibitory Factor (LIF, 100 ng/ml) for 2 h or 15 min, respectively. STAT3 dimers translocate to the nucleus of stimulated cells, as expected (Scale bar, 20µm). C, Immunoblotting of STAT3 in nuclear extracts from HEK293 or HeLa cells transfected with Venus-STAT3 constructs and incubated in the presence or absence of LIF (100 ng/ml) for 2 h or 15 min, respectively. Three bands are observed, corresponding to V1-STAT3 (1), V2-STAT3 (2) and endogenous STAT3 (E). D, LIF does not induce further STAT3 dimerization, as determined by flow cytometry. The histogram (right) shows that there is almost perfect overlap between the fluorescent signal emitted by cells in the presence or absence of LIF

**Figure S2.** The disease-associated L78R mutation inhibits STAT3 dimerization, and promotes its aggregation and nuclear translocation. A, HeLa cells were co-transfected with several BiFC combinations: the wild type (WT) STAT3 pair (control), or WT STAT3 with i) the L78R STAT3 mutant (since L78R is a somatic, dominant STAT3 mutation), or ii) Mdm2 or p53, two proteins that in principle should not interact with STAT3 as negative controls. B, Representative cells for the WT STAT3 pair and the combination containing the L78R mutant. While most cells transfected with the WT STAT3 pair show a predominant cytoplasmic localization of the dimers, the introduction of the L78R mutation changes the distribution pattern towards the production of cytoplasmic inclusions and a predominantly nuclear location. C, Quantification of microscopy pictures. As explained in the main text, we classified cells according to the relative intensity and the location of the fluorescence signal in five categories: cytoplasmic, nuclear, both cytoplasmic and nuclear, mitochondrial and inclusions. Data are shown as the average ± SEM of n=12 (Wild-type, WT) or n=3 (L78R) independent experiments. *, significant vs the symmetric Wild-type STAT3 pair, p<0.05

**Figure S3.** Selected PTMs (symmetric or asymmetric) do not regulate STAT3 homodimerization in unstimulated cells. The graph represents the percentage of fluorescent cells observed in each Venus-STAT3 BiFC combination by means of flow cytometry. The numeric code is represented in the table below the graph. Errors represent the Standard Error. Statistical analysis was carried out by means of a One-way ANOVA. No combination produced significant changes

**Figure S4.** Examples of cells showing mitochondrial localization of STAT3 dimers versus STAT3 inclusions. Mitochondrial localization of STAT3 dimers was monitored by co-localization of fluorescent foci with Mitotracker Red. When there were fluorescent foci that did not co-localize with Mitotracker Red we classified them as “Inclusions”
